# Using Deep Transfer Learning to Detect Hyperkalemia From Ambulatory Electrocardiogram Monitors in Intensive Care Units: Personalized Medicine Approach

**DOI:** 10.2196/41163

**Published:** 2022-12-05

**Authors:** I-Min Chiu, Jhu-Yin Cheng, Tien-Yu Chen, Yi-Min Wang, Chi-Yung Cheng, Chia-Te Kung, Fu-Jen Cheng, Fei-Fei Flora Yau, Chun-Hung Richard Lin

**Affiliations:** 1 Department of Computer Science and Engineering National Sun Yat-sen University Kaohsiung City Taiwan; 2 Department of Emergency Medicine Kaohsiung Chang Gung Memorial Hospital Kaohsiung City Taiwan; 3 Division of Cardiology Department of Internal Medicine Kaohsiung Chang Gung Memorial Hospital Kaohsiung City Taiwan

**Keywords:** deep learning, transfer learning, hyperkalemia, electrocardiogram, ECG monitor, ICU, personalized medicine

## Abstract

**Background:**

Hyperkalemia is a critical condition, especially in intensive care units. So far, there have been no accurate and noninvasive methods for recognizing hyperkalemia events on ambulatory electrocardiogram monitors.

**Objective:**

This study aimed to improve the accuracy of hyperkalemia predictions from ambulatory electrocardiogram (ECG) monitors using a personalized transfer learning method; this would be done by training a generic model and refining it with personal data.

**Methods:**

This retrospective cohort study used open source data from the Waveform Database Matched Subset of the Medical Information Mart From Intensive Care III (MIMIC-III). We included patients with multiple serum potassium test results and matched ECG data from the MIMIC-III database. A 1D convolutional neural network–based deep learning model was first developed to predict hyperkalemia in a generic population. Once the model achieved a state-of-the-art performance, it was used in an active transfer learning process to perform patient-adaptive heartbeat classification tasks.

**Results:**

The results show that by acquiring data from each new patient, the personalized model can improve the accuracy of hyperkalemia detection significantly, from an average of 0.604 (SD 0.211) to 0.980 (SD 0.078), when compared with the generic model. Moreover, the area under the receiver operating characteristic curve level improved from 0.729 (SD 0.240) to 0.945 (SD 0.094).

**Conclusions:**

By using the deep transfer learning method, we were able to build a clinical standard model for hyperkalemia detection using ambulatory ECG monitors. These findings could potentially be extended to applications that continuously monitor one’s ECGs for early alerts of hyperkalemia and help avoid unnecessary blood tests.

## Introduction

Hyperkalemia is a metabolic condition that contributes to more than 800,000 emergency department visits in the United States annually [1]. It is associated with life-threatening ventricular arrhythmias and sudden cardiac arrest, and it is especially common among patients with chronic kidney disease due to their impaired renal potassium homeostasis and long-term use of renin-angiotensin-aldosterone system inhibitors [2,3].

Patients under critical care may receive regular blood tests for electrolytes every few hours or days [4]. Many potential factors can affect potassium levels in between monitoring periods, such as diets, metabolic acidosis, and alterations in the intracellular/extracellular potassium distribution. Therefore, noninvasive monitoring techniques of potassium levels can help fill the gap between blood tests for early detection of this potentially deadly condition.

It is well known that a variety of changes on the electrocardiogram (ECG) can be associated with hyperkalemia, including but not limited to peaked T waves, shortened QT interval, lengthening of PR interval, and QRS duration [5]. Accurate human interpretations of these ECG patterns requires a steep learning curve, and the sensitivity of physician diagnoses has been estimated to be as low as 34% to 43% [6], not to mention the impossibility of self-detection of hyperkalemia using only symptoms and signs. There have been several successes in leveraging deep learning models to detect electrolyte abnormalities on ECGs [7-10], and previous studies have proven the feasibility of this approach for detecting subtle signals from ECGs. However, low specificity and a high false-positive rate could cause alert fatigue among physicians and anxiety in patients.

A recent study exploring a personalized deep learning–based system to detect hypoglycemia via ECG data has yielded promising results [11]. The study collected dozens of personal blood glucose and corresponding ECG data and adopted a convolution neural network to develop a personalized deep learning model to predict the hypoglycemia event. Since it is nearly impossible to gather enough personal data in a real-world setting, our study proposes a personalized transfer learning method by first training a general model and then refining it with personal data to improve the accuracy of hyperkalemia predictions, diminish the intersubject heterogeneity, and advance toward personalized medicines.

## Methods

### Ethics Approval

The data collection and study protocols were approved by the Institutional Review Board of Chang Gung Medical Foundation (202001217B0; date of approval July 21, 2020). The study was conducted following the standards issued by the World Medical Association’s Declaration of Helsinki. The data that support the findings of this study are openly available in the Medical Information Mart From Intensive Care III (MIMIC-III) Waveform Database Matched Subset [12,13].

### Data Set Collection

This study used data from the Waveform Database Matched Subset. The data set contains 22,317 waveform records and 22,247 numeric records for 10,282 distinct intensive care unit (ICU) patients who were admitted to critical care units of medical centers in the United States between 2001 and 2012. These recordings typically include digitized signals, such as ECG, arterial blood pressure, and respiration; additionally, they include periodic measurements such as heart rate, oxygen saturation, and blood pressure. The data set’s ECG signals were usually of leads I, II, or V. This subset represents records for which the patients have been identified and whose corresponding clinical records are available in its matched clinical database.

### Patient Population

All patients with a plasma potassium level during admission, from the MIMIC-III data set, were included. However, patients without lead II ECG signals at the time of the potassium level test and patients with atrial fibrillation, pacing rhythm, or other medical conditions for which a complete heartbeat cycle could not be distinctly identified in the ECG were excluded.

This study aimed to distinguish hyperkalemia from a normal level based on ECG features. Patients with at least 8 records of hyperkalemia and normokalemia each were adopted for personalized transfer learning. The others were selected for generic model training.

### Data Preprocessing

ECG excerpts from 10 minutes before the time of serum potassium tests were annotated as corresponding to hyperkalemia or normokalemia according to the test results. Hyperkalemia was defined as serum potassium concentration values above 5.5 mEq/L and normokalemia as serum concentration between 3.5 mEq/L and 5 mEq/L. We excluded serum potassium levels between 5 and 5.5 mEq/L to ensure that no consecutive heartbeats would be considered as both hyper and normal, therefore reducing overfitting of the model.

After collection, ECG excerpts were filtered using finite impulse response techniques and underwent manual inspection to exclude those containing too much ECG signal noise. This process helps to reduce overfitting of the model and deviating to noisy data.

After retrieving the corresponding ECG signals, each ECG record was segmented into heartbeats of 120 samplings based on the fiducial point, which was the R peak. Each heartbeat segment contained 40 samples preceding the R peak and 80 samples after, in which the R peak was the 41st sample.

### Generic Model Training

The goal was to train a generic model using a large set of heartbeats and to leverage a transfer learning algorithm that could refine the generalized model into a subject-specific model. The purpose of training the deep learning model during this step was to obtain a pretrained weight for transfer learning, as it helps the model to learn the shape of the ECG features for hyperkalemia.

All the data were randomly split into a training set, validation set, and test data set in a 6:2:2 ratio. This study used the ResNet architecture as the baseline architecture [14]. ResNet stands for residual network; it is an innovative neural network first introduced in 2015 that won the top position at the ImageNet classification competition, with an error of only 3.57%. The ResNet structure is widely used in ECG classification tasks since the residual block component allows the model to add more layers that help to detect the complex pattern of ECG morphology. In 2019, a study demonstrated a cardiologist-level arrhythmia detection task in ambulatory ECGs using a 1D ResNet model [15]. Since ECG data in the MIMIC-III database is 1D, we substituted the 2D convolution layers with 1D for detailed feature extraction. Besides that, we did not change the size of the convolutional block, stride, and number of filters. After training, model weights for the best performance in the validation set were saved as pretrained for personal transfer learning.

### Personalized Transfer Learning

Transfer learning applies knowledge obtained by solving one problem to a related problem. The general procedure for transfer learning is to pretrain a deep learning model with a large data set (eg, generic population for hyperkalemia), then refine it using a much smaller target data set (eg, personal data for hyperkalemia).

In personalized training steps, we adopted the same architecture of the 1D ResNet model in the general population for allowing the weight to be preserved [16]. Before refining it with personal data, we replaced its classification layer with a fully connected layer, the weights of which were randomly initialized. We froze the pretrained weights in the first few blocks of the convolution layers and trained the last few blocks of layers for 5 epochs using personal ECG data.

For each patient in the personalized group, 25% of potassium records and their corresponding ECG data were preserved as the test data set. During the training process, ECG data representing one record each of hyperkalemia and normokalemia were used as inputs. The training process continued for five rounds at most, depending on the total number of potassium records for each patient. The model performance after each training process was assessed to measure the performance changes. The deep learning models were trained with the TensorFlow application programming interface on the Google Colab platform.

### Statistical Analysis

Continuous variables with normal distribution were presented either as means (SDs) or medians (IQRs). Continuous variables were analyzed using the Mann-Whitney *U* test, and the final model was validated using a majority voting scheme that runs through all the single heartbeats in a 10-minute ECG strip to determine the prediction result. All performance predictions were assessed using accuracy, area under the receiver operating characteristic curve (AUC), sensitivity, and specificity. All statistical analyses were performed on SPSS 26 for Mac (IBM Corp).

## Results

### Characteristics of Data Sets

**Figure 1:**
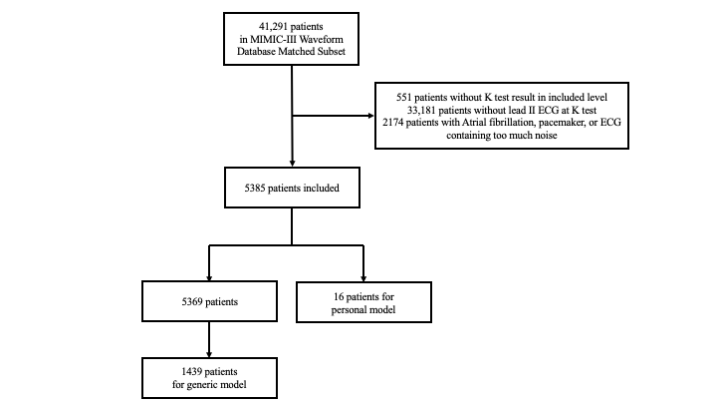
Patient inclusion flowchart. ECG: electrocardiogram; MIMIC-III: Medical Information Mart From Intensive Care III.

In this study, of the 41,291 patients in the MIMIC-III database, 5385 who fulfilled the criteria were included for analysis; 16 were chosen for personalized model development and validation and 5369 for pretrained general model development. To avoid deviation of general model prediction toward normokalemia, balanced ECG samples of hyperkalemia and normokalemia retrieved from 1439 patients were used. These included 1341 hyperkalemia records of 721 patients and 1325 normokalemia records of 718 patients. The inclusion flowchart is shown in Figure 1. Demographics and clinical characteristics of the two development populations are shown in Table 1. For the personal model development, patients’ median age was 50 (IQR 43-60) years, and 13 (81%) of them were male. Concerning ethnic groups, 7 (44%) patients and 3 (19%) patients were White and African American, respectively. The mean serum potassium levels of hyperkalemia and normokalemia were 6.3 (SD 0.64) mEq/L and 4.3 (SD0.40) mEq/L and 6.2 (SD 0.70) mEq/L and 4.1 (SD 0.44) mEq/L in the general and personalized groups, respectively.

**Table 1:** Patient demographic in generic population and personal population.

Variables		Generic population (n=1439)	Personal population (n=16)
Age (years), median (IQR)		64 (52-76)	50 (43-60)
**Gender, n (%)**			
	Male	610 (59.6)	13 (81.2)
	Female	413 (40.4)	3 (18.8)
**Ethnicity, n (%)**			
	White	907 (63.0)	7 (43.8)
	African American	176 (12.2)	3 (18.8)
	Hispanic	71 (4.6)	0 (0.0)
	Asian	45 (3.1)	1 (6.2)
	American Indian	6 (0.4)	0 (0.0)
	Other	50 (3.5)	1 (6.2)
	Unknown	184 (12.8)	4 (25.0)
**Body index, median (IQR)**			
	Height (cm)	170.0 (160.2-178.0)	176.5 (169.2-186.8)
	Weight (kg)	79.2 (66.9-94.6)	89.2 (71.4-108.3)
**Serum level (mEq/L), mean (SD)**			
	Normokalemia	4.3 (0.40)	4.1 (0.44)
	Hyperkalemia	6.3 (0.64)	6.2 (0.70)

### Development of a Generic Model

**Figure 2:**
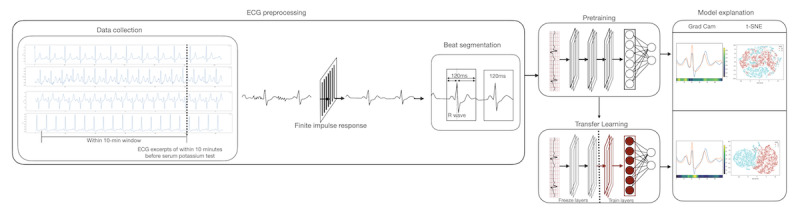
Proposed transfer learning algorithm for recognizing hyperkalemia from ambulatory ECG monitoring. ECG: electrocardiogram; Grad Cam: gradient-weighted class activation mapping; t-SNE: t-distributed stochastic neighbor embedding.

The proposed transfer learning method is depicted in Figure 2. For generic model development, in the training set, 152,322 and 172,388 normokalemic and hyperkalemic heartbeats, respectively, were contributed by 881 patients. In the validation set, 51,468 normokalemic and 53,488 hyperkalemic heartbeats were segmented from 280 patients. In the test set, 52,134 normokalemic and 53,412 hyperkalemic heartbeats were present in 278 patients.

This study adopted the ResNet-50 model for training the classification task. ResNet-50 is a network that has 50 layers in depth. Rather than its shallow version, ResNet-18 or ResNet-34, ResNet-50 combines the structure of residual and bottleneck blocks in the convolutional layer to reduce computing resources. Before each convolutional layer, we applied batch normalization and a rectified linear activation, adopting the original design of the preactivation block. The network was trained with random initialization of the weights. We used the Adam optimizer with the default parameters and a mini-batch size of 1024. We initialized the learning rate to 5 × 10^–5^ and reduced it by a factor of 2 when the developmentally set loss stopped improving for 3 consecutive epochs. The model was trained for 50 epochs using the training set. We saved the model that achieved minimal loss in the validation set during training as the generic, or the so-called pretrained, model. The final pretrained model’s prediction accuracy was 0.724, 0.639, and 0.627 in the training, validation, and test sets, respectively.

### Development of a Personalized Model

The complete list of patients included for personalized model development is shown in Multimedia Appendix 1. Before starting the personalized training process, the performance of the pretrained model was assessed on each patient to obtain baseline metrics. The same convolutional neural network (CNN) architecture as ResNet-50 was used, the pretrained weight in the first several layers were frozen, and the model was fine-tuned in each training round. The improvement in predictions after each training round for all personalized group patients are shown in Multimedia Appendix 2.

**Figure 3:**
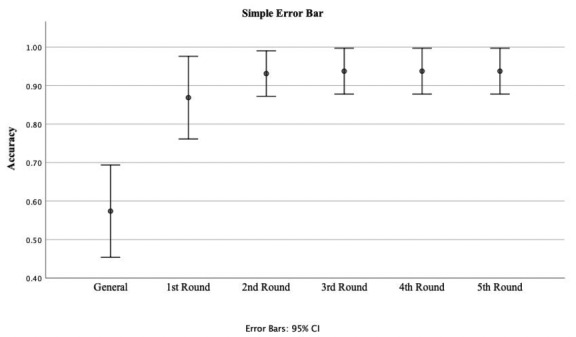
Accuracy improvement on number of personalized training rounds.

On average, accuracy improved from 0.604 (SD 0.211) to 0.895 (SD 0.189; *P*<.001) after the first round of training (Figure 3) and achieved a plateau at 0.942 (SD 0.104) after the second round of training. In addition, the AUC level improved from an average of 0.729 (SD 0.240) to 0.918 (SD 0.149) after the first round of training and continued to increase after the second round to 0.945 (SD 0.094), being maintained at that level thereafter. After five rounds of training, the personalized model was able to predict hyperkalemia with an average accuracy of 0.980 (SD 0.078). Moreover, the sensitivity and specificity of model prediction improved after personalized transfer learning. There was a significant increase in average sensitivity after the second round of personalized transfer learning (0.674, SD 0.456 vs 0.953, SD 0.160; *P*=.03) and improvement in average specificity after the first round of personalized transfer learning (0.628, SD 0.417 vs 0.907, SD 0.321; *P*=.03; Multimedia Appendix 2).

### Model Interpretation and Visualization

**Figure 4:**
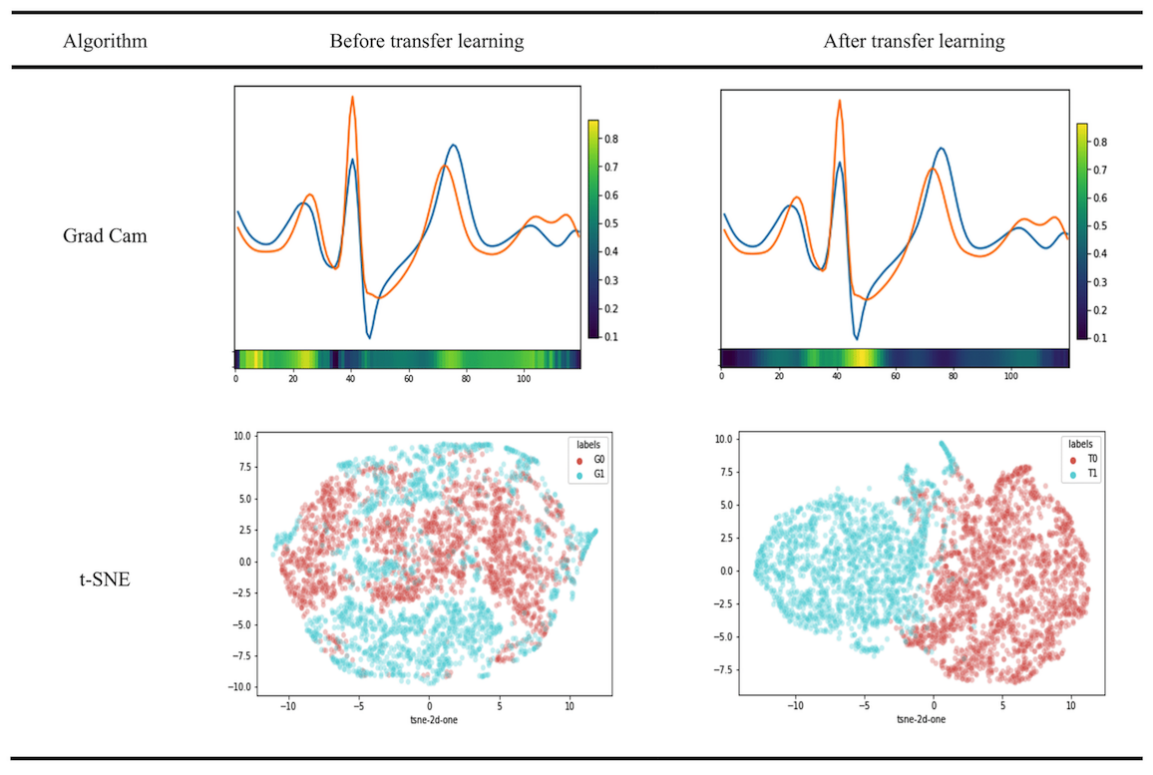
Visualization of model prediction before and after personalized transfer learning. Grad Cam: gradient-weighted class activation mapping; t-SNE: t-distributed stochastic neighbor embedding.

After refining the model for each candidate, we generated the gradient-weighted class activation mapping (Grad Cam) for obtaining visual explanations from the model [17]. We selected one patient for demonstration (Figure 4). The blue and orange lines represented the average plot of segmented heartbeats from hyperkalemia and normokalemia in the test set of that patient, respectively. Grad Cam uses the gradients of the final convolutional layer to produce a coarse localization map, highlighting important regions in the image for predicting the concept. We overlapped the activation maps on the original ECG reading to highlight the emphasized areas in the CNN. In this patient, the region of interest was dispersed before transfer learning and thereafter became more focused over the QRS segment.

To further address the effect of personalized transfer learning and to visualize the learned embeddings [18], we used the t-distributed stochastic neighbor embedding (t-SNE) method that extracted features from the last convolutional layer and converted these high-dimension features to 2D features, which we could analyze using the scatter plot.

This study presents a visualization of the same patient’s heartbeats using a t-SNE scatter plot and Grad Cam (Figure 4). In the figure, the light blue dot represents the hyperkalemia ECG data and the red dot the normokalemia data. It can be observed that the dots presenting each heartbeat corresponding to the hyperkalemia and normokalemia were quite muddled at the beginning and gradually organized into two clusters that were easier to separate by a straight line in a 2D space after personalized transfer learning. The t-SNE plots imply that after transfer learning, it is easier to distinguish the two classes clearly through the CNN model.

## Discussion

### Principal Findings

Our study showed the feasibility of detecting hyperkalemia by using transfer learning on personalized single-lead ECG readings. In previous research, one of the biggest challenges of deep learning for ECG classification was the scalability of a single model in different populations. The reasons for this generalization problem included individually varying ECG signal properties that depended on various factors such as weight, height, age, and physical conditions [19], not to mention data collected from different institutions and by other technicians. Therefore, expecting a generalized framework to be functional for the general population can be problematic [20]. The novelty of this study is that it considers the same classification task in the generic and personal predictions as different domains, which is a novel area in dyskalemia prediction using ECGs. Since other medical conditions may affect patients’ ECG, the ECG signal of hyperkalemia is not specific. Patients’ different medical diseases contribute to how their ECGs manifest. Due to large interpersonal variation, we believe that one generic model cannot cover a variety of illnesses that affect ECG signals. By using personalized transfer learning, we can load personal information into the model and make it better fit the personal ECG change.

In our study, when comparing the results with our pretrained model, transfer learning on personalized data showed a considerable increase in accuracy and AUC, and significantly boosted both sensitivity (mean 0.674, SD 0.456 vs mean 0.953, SD 0.160; *P*=.03) and specificity (mean 0.628, SD 0.417 vs mean 0.907, SD 0.321; *P*=.03), which demonstrated a good precision by ambulatory ECG monitors.

Previous studies applying deep learning to ECG classification focused on arrhythmia detection [21-23]. One of the reasons may be the availability of benchmark data sets that have reliable annotations that are mostly limited to arrhythmia, such as atrial fibrillation. Within the deep learning arrhythmia detection domain, transfer learning had been extensively explored to enhance the performance of CNNs; in a study, Weimann and Conrad [24] showed that transfer learning effectively reduces the number of annotations required to achieve the same performance as CNNs that are not pretrained. However, apart from arrhythmias, many other metabolic conditions such as hyperkalemia, hypokalemia, and hypomagnesemia can also be manifested in ECG readings [25]; hence, successes in arrhythmia detection should be expandable for detection of metabolic diseases through ECGs. In 2019, a study by Galloway et al [10] used 2 to 4 leads of the 12 leads of an ECG to develop a deep learning model to predict hyperkalemia and demonstrated a sensitivity of 88.9%-91.3% and a specificity of 54.7%-63.2%. The study proved that screening for hyperkalemia in patients with chronic kidney disease was feasible. A recent study using all 12 leads from complete ECGs to predict both hyperkalemia and hypokalemia showed better results with a balanced accuracy of around 79.9%-82.8% [26]. Nevertheless, a higher standard of prediction performance may be desired to aid in clinical practice.

Personalized medicine, mostly discussed in genomic research, refers to the application of specific patient information to make a more informed choice regarding their optimal therapeutic treatments or precise diagnoses, rather than relying on population-based trends [27]. Recent studies have shown that a personalized medicine approach could benefit disease diagnosis with ECGs. A 2018 study proved that by acquiring about 5% of personal ECG data from each new patient, the personalized deep learning model was able to substantially improve the precision of disease detection in contrast with the generic model [28]. Another study using a personalized deep learning system to detect hypoglycemic events from ECG rhythms found that the model overcame the limitations of intersubject variability in conventional systems [11]. The concept of adopting a personalized approach on ECG interpretation could improve specificity, which could prevent alert fatigue and overinterventions in a real-world setting. Our study demonstrated that transfer learning through a personalized approach required fewer ECG data queries and could bring about substantial improvements on sensitivity and specificity, which are useful in minimizing false alerts.

To enhance the interpretability of our model, we applied the Grad Cam method that allows one to easily scrutinize the areas the model is relying on (Figure 4). Between our generalized model and personalized model trained from transfer learning, the former focuses more on broad characteristics such as the P, QRS, and T segments that were considered as a well-defined series of changes by previous literature [29]; the latter focused more on a few localized areas that we believe are related to interpersonal differences. In each patient in the personalized group, the highlighted area of the ECG section from the Grad Cam visualization was different from the others. This explains how the personalized model performed better than the generic model and the interpersonal variation of ECGs occurred according to the level of hyperkalemia.

We used the t-SNE method for visualizing our learned embeddings in a lower dimension. This particular approach finds a joint probability distribution in a low dimension that closely represents the data points in the original high dimension by using gradient descent [18]. From our t-SNE results regarding the last convolution layer of the deep learning model, the personalized model showed two discrete groups compared to a more heterogeneous appearance in the generalized model (Figure 4). This shows that learned embedding can better separate the heartbeats according to potassium levels.

The study results demonstrate the potential of leveraging transfer learning on a personalized data set with fewer data while producing comparable results. Even in an individual participant, by using only four sets of data, the AUC increased significantly and many even plateaued. However, our study did have limitations that warrant future investigations and validations. First, since its database came from ICU records, only 16 patients who had multiple potassium drawings and corresponding ECG readings were included. However, in reality, it is difficult for a single patient to undergo so many blood tests, the results of each having its own corresponding ECG data. Second, by extending the framework to be multimodal, including other physiological signals such as blood pressure, age, gender, underlying disease, and weight, we could further enhance our model’s performance, and this should, therefore, be the future research goal.

### Conclusion

Using personalized transfer learning on single-lead ECG readings is sufficient to yield high AUC and accurate results for hyperkalemia detection. The visualization of the model interpretation demonstrated the interpersonal differences on ECG change according to hyperkalemia. This finding could potentially be extended to applications that continuously monitor one’s ECGs, thus serving as a surveillance system for patients at high risk of hyperkalemia and avoiding unnecessary blood tests.

## Appendix

Multimedia Appendix 1 Complete list of patients included for the personalized model development.

Multimedia Appendix 2 The improvement in predictions after each training round for all patients in the personalized group.

## Abbreviations

AUC: area under the receiver operating characteristic curve
CNN: convolutional neural network
ECG: electrocardiogram
Grad Cam: gradient-weighted class activation mapping
ICU: intensive care unit
MIMIC-III: Medical Information Mart From Intensive Care III
t-SNE: t-distributed stochastic neighbor embedding

## Data Availability

The data that support the findings of this study are openly available in the Medical Information Mart From Intensive Care III Waveform Database Matched Subset [13].
