# Supplementary material for: Using Deep Transfer Learning to Detect Hyperkalemia From Ambulatory Electrocardiogram Monitors in Intensive Care Units: Personalized Medicine Approach
Source: J Med Internet Res. 2022 Dec 5;24(12):e41163. doi: 10.2196/41163 (PMC9764151; doi:10.2196/41163)
Supplement: Multimedia Appendix 1 [file jmir_v24i12e41163_app1.pdf]

Appendix 1. Table 1 that demonstrates the complete list of patients included for personalized model development

Subject_ID	HADM_ID	gender	height(cm)	weight(kg)	age	ethnicity	ICD_9 Code1	ICD_9 Code2	ICD_9 Code3	ICD_9 Code4	ICD_9 Code5
13593	113326	F	165	62.0	58	BLACK/AFRICAN AMERICAN	03811	51881	78552	5856	6821
18681	187798	M		71.4	31	BLACK/AFRICAN AMERICAN	570	2867	042	78551	40391
32084	178430	M	172.72	81.3	44	WHITE	41071	9971	2851	41401	2767
41619	136884	M	173	93.8	49	WHITE	96905	51881	5845	5070	43411
42530	145307	M	180	68.1	49	UNKNOWN/NOT SPECIFIED	0389	78552	51853	5100	486
46092	129119	M	168	69.4	79	ASIAN	0388	570	78552	51881	48282
48388	156310	M	188	89.2	58	WHITE	56211	0380	41511	4821	99591
65112	147914	M	183	87.0	26	UNABLE TO OBTAIN	85185	0389	78552	3485	570
68127	183283	M			58	OTHER	25040	40391	41189	2851	99681
73595	198022	M	193	110.0	48	UNKNOWN/NOT SPECIFIED	4210	41071	5185	78551	5856
75350	171975	M	183	100.0	51	WHITE	9001	570	8601	5845	53140
78076	138363	M	163	108.3	70	WHITE	5770	53784	5070	51881	56738
81475	101662	M	203	91.7	61	BLACK/AFRICAN AMERICAN	51881	5570	49322	99812	9971
81593	192553	F		118.1	68	WHITE	99931	03812	51881	78552	5845
83608	149069	F		124.0	42	UNABLE TO OBTAIN	57420	51881	5845	486	5070
89437	147128	M	173	85.0	41	WHITE	0279	0388	51881	78552	5845
