# Supplementary material for: Using Deep Transfer Learning to Detect Hyperkalemia From Ambulatory Electrocardiogram Monitors in Intensive Care Units: Personalized Medicine Approach
Source: J Med Internet Res. 2022 Dec 5;24(12):e41163. doi: 10.2196/41163 (PMC9764151; doi:10.2196/41163)
Supplement: Multimedia Appendix 2 [file jmir_v24i12e41163_app2.pdf]

Appendix 2. Table 2 that demonstrates the improvement in predictions after each training round for all patients in the personalized group.

| Parameters | General | 1st round | p-value | 2nd round | p-value | 3rd round | p-value | 4th round | p-value | 5th round | p-value |
|---|---|---|---|---|---|---|---|---|---|---|---|
| Accuracy | 0.604±0.211 | 0.895±0.189 | <0.001 | 0.942±0.104 | <0.001 | 0.951±0.105 | <0.001 | 0.951±0.105 | <0.001 | 0.980±0.078 | <0.001 |
| AUC | 0.729±0.189 | 0.918±0.142 | <0.001 | 0.939±0.121 | <0.001 | 0.945±0.109 | <0.001 | 0.953±0.097 | <0.001 | 0.953±0.097 | <0.001 |
| Sensitivity | 0.674±0.456 | 0.884±0.270 | 0.259 | 0.953±0.160 | 0.027 | 0.951±0.160 | 0.029 | 0.951±0.160 | 0.029 | 0.938±0.156 | 0.044 |
| Specificity | 0.628±0.417 | 0.907±0.321 | 0.029 | 0.930±0.160 | 0.012 | 0.951±0.160 | 0.006 | 0.951±0.160 | 0.021 | 1.0 | <0.001 |
